# Dual checkpoint targeting of B7-H3 and PD-1 with enoblituzumab and pembrolizumab in advanced solid tumors: interim results from a multicenter phase I/II trial

**DOI:** 10.1136/jitc-2021-004424

**Published:** 2022-04-12

**Authors:** Charu Aggarwal, Amy Prawira, Scott Antonia, Osama Rahma, Anthony Tolcher, Roger B Cohen, Yanyan Lou, Ralph Hauke, Nicholas Vogelzang, Dan P Zandberg, Arash Rezazadeh Kalebasty, Victoria Atkinson, Alex A Adjei, Mahesh Seetharam, Ariel Birnbaum, Andrew Weickhardt, Vinod Ganju, Anthony M Joshua, Rosetta Cavallo, Linda Peng, Xiaoyu Zhang, Sanjeev Kaul, Jan Baughman, Ezio Bonvini, Paul A Moore, Stacie M Goldberg, Fernanda I Arnaldez, Robert L Ferris, Nehal J Lakhani

**Affiliations:** 1 Abramson Cancer Center, University of Pennsylvania, Philadelphia, Pennsylvania, USA; 2 Kinghorn Cancer Centre, St. Vincent’s Hospital, Sydney, New South Wales, Australia; 3 Duke Cancer Institute Center for Cancer Immunotherapy, Durham, North Carolina, USA; 4 Moffitt Cancer Center, Tampa, Florida, USA; 5 Dana-Farber Cancer Institute, Boston, Massachusetts, USA; 6 NEXT Oncology, San Antonio, Texas, USA; 7 START-South Texas, San Antonio, Texas, USA; 8 Mayo Clinic, Jacksonville, Florida, USA; 9 Nebraska Cancer Specialists, Omaha, Nebraska, USA; 10 Comprehensive Cancer Centers of Nevada, Las Vegas, Nevada, USA; 11 UPMC Hillman Cancer Center, Pittsburgh, Pennsylvania, USA; 12 University of Maryland Marlene and Stewart Greenebaum Comprehensive Cancer Center, Baltimore, Maryland, USA; 13 Norton Cancer Institute, Louisville, Kentucky, USA; 14 Princess Alexandra Hospital, Woolloongabba, Queensland, Australia; 15 Mayo Clinic, Rochester, Minnesota, USA; 16 Mayo Clinic, Scottsdale, Arizona, USA; 17 Rhode Island Hospital, Providence, Rhode Island, USA; 18 Austin Health, Heidelberg, Victoria, Australia; 19 Peninsula and Southeast Oncology, Frankston, Victoria, Australia; 20 MacroGenics, Inc, Rockville, Maryland, USA; 21 AstraZeneca, Gaithersburg, Maryland, USA; 22 START Midwest, Grand Rapids, Michigan, USA

**Keywords:** head and neck neoplasms, lung neoplasms, clinical trials as topic, drug therapy, combination, immunotherapy

## Abstract

**Background:**

Availability of checkpoint inhibitors has created a paradigm shift in the management of patients with solid tumors. Despite this, most patients do not respond to immunotherapy, and there is considerable interest in developing combination therapies to improve response rates and outcomes. B7-H3 (CD276) is a member of the B7 family of cell surface molecules and provides an alternative immune checkpoint molecule to therapeutically target alone or in combination with programmed cell death-1 (PD-1)–targeted therapies. Enoblituzumab, an investigational anti-B7-H3 humanized monoclonal antibody, incorporates an immunoglobulin G1 fragment crystallizable (Fc) domain that enhances Fcγ receptor-mediated antibody-dependent cellular cytotoxicity. Coordinated engagement of innate and adaptive immunity by targeting distinct members of the B7 family (B7-H3 and PD-1) is hypothesized to provide greater antitumor activity than either agent alone.

**Methods:**

In this phase I/II study, patients received intravenous enoblituzumab (3–15 mg/kg) weekly plus intravenous pembrolizumab (2 mg/kg) every 3 weeks during dose-escalation and cohort expansion. Expansion cohorts included non–small cell lung cancer (NSCLC; checkpoint inhibitor [CPI]–naïve and post-CPI, programmed death-ligand 1 [PD-L1] <1%), head and neck squamous cell carcinoma (HNSCC; CPI-naïve), urothelial cancer (post-CPI), and melanoma (post-CPI). Disease was assessed using Response Evaluation Criteria in Solid Tumors version 1.1 after 6 weeks and every 9 weeks thereafter. Safety and pharmacokinetic data were provided for all enrolled patients; efficacy data focused on HNSCC and NSCLC cohorts.

**Results:**

Overall, 133 patients were enrolled and received ≥1 dose of study treatment. The maximum tolerated dose of enoblituzumab with pembrolizumab at 2 mg/kg was not reached. Intravenous enoblituzumab (15 mg/kg) every 3 weeks plus pembrolizumab (2 mg/kg) every 3 weeks was recommended for phase II evaluation. Treatment-related adverse events occurred in 116 patients (87.2%) and were grade ≥3 in 28.6%. One treatment-related death occurred (pneumonitis). Objective responses occurred in 6 of 18 (33.3% [95% CI 13.3 to 59.0]) patients with CPI-naïve HNSCC and in 5 of 14 (35.7% [95% CI 12.8 to 64.9]) patients with CPI-naïve NSCLC.

**Conclusions:**

Checkpoint targeting with enoblituzumab and pembrolizumab demonstrated acceptable safety and antitumor activity in patients with CPI-naïve HNSCC and NSCLC.

**Trial registration number:**

NCT02475213.

**Key messages**

**What is already known on this topic**

- Enoblituzumab, an anti-B7-H3 monoclonal antibody is well tolerated and associated with encouraging tumor shrinkage in patients with non–small cell lung cancer (NSCLC), head and neck squamous cell carcinoma (HNSCC), melanoma, and bladder cancer on a phase I trial.
- We hypothesized that combination therapy of enoblituzumab and pembrolizumab would result in coordinated engagement of innate and adaptive arms of the immune system to maximize tumor regression in select solid tumors.

**What this study adds**

- The results of this phase I/II study demonstrate that dual immunotherapy with the combination of enoblituzumab and pembrolizumab have an acceptable safety profile and robust activity in patients with checkpoint inhibitor-naïve metastatic NSCLC (36% overall response rate [ORR]) and recurrent/metastatic HNSCC (33% ORR).

**How this study might affect research, practice, or policy**

- Further studies are required to confirm these preliminary results and to eventually compare efficacy and toxicity outcomes of this dual immunotherapy with other currently approved immunotherapy-based combinations in patients with advanced CPI-naïve NSCLC and HNSCC.

## Introduction

The availability and approval of immune checkpoint inhibitors (CPIs), particularly antibodies blocking the programmed cell death (PD)-1 pathway, have changed the treatment paradigm for multiple advanced solid malignancies, including melanoma, non–small cell lung cancer (NSCLC), head and neck squamous cell carcinoma (HNSCC), and bladder cancer. While only 20%–40% of patients derive benefit from immunotherapy,^1^ however, and there remains considerable interest in developing combination therapies to improve response rates and outcomes. Combination chemotherapy with PD-1 inhibition has emerged as a promising approach for patients with NSCLC, gastric, and, to a lesser extent, HNSCC.^2–6^ However, similar benefit from combining chemotherapy with immunotherapy has not been demonstrated in other malignancies.^6^ The toxicity profile observed from the combination of chemotherapy and immunotherapy underscores the continued need for safer combination regimens with improved tolerability. Dual checkpoint blockade with PD-1 and cytotoxic T-lymphocyte antigen (CTLA)-4 inhibition is another approach that has been evaluated in NSCLC, RCC, and melanoma. This approved combination is associated with improved response rates compared with CPI monotherapy, but there is a high rate of treatment-related adverse events (TRAEs, 96% all grades and 55% grade 3/4) and immune-related adverse events (irAEs, 88% all grades and 40% grade 3/4).^7^

B7-H3 (CD276) is a member of the B7 family of cell surface molecules that also includes CTLA-4 ligands B7-1 (CD80) and B7-2 (CD86) and programmed death-ligands, PD-L1 (B7-H1) and PD-L2 (B7DC). B7-H3 provides an alternative immune checkpoint molecule to therapeutically target alone or in combination with PD-1-targeted therapies.^8–10^ B7-H3 expression has been demonstrated on antigen-presenting cells (APC), but it is limited or absent in most normal tissue.^11^ In contrast, high B7-H3 expression is observed on solid tumors, with expression detectable on malignant epithelial cells, the tumor vasculature, and subsets of infiltrating immune cells. While the receptor for B7-H3 is unknown, numerous immunomodulatory roles, predominantly inhibitory, have been described for B7-H3.^11 12^ In addition to its role in immune modulation, B7-H3 also promotes pro-tumorigenic functions such as tumor migration, invasion, metastases, resistance, and metabolism.^11 12^

B7-H3 has been shown to inhibit T-cell activation and cytokine production,^13 14^ and B7-H3 expression on tumor cells is generally thought to convey protection from immune attack by natural killer (NK) and cytotoxic T cells. Consistent with its potential role in mediating tumor immune evasion, B7-H3 expression is associated with adverse clinical outcomes, including poor survival across multiple cancer types including NSCLC and HNSCC.^15 16^ In murine tumor models, the combination of anti-B7-H3 and PD-1 pathway-targeting monoclonal antibodies (mAbs) provides greater antitumor control than that achieved by either agent alone, suggesting independent pathways of tumor immune evasion.^9 10^ The observation that therapeutic response to anti-PD-1 therapy is relatively limited in patients with NSCLC expressing B7-H3 compared with the higher response rate observed in those who are B7-H3 negative provides further support to evaluate combined targeting of B7-H3 and PD-1.^9^

The broad surface expression of B7-H3 on many malignant neoplasms, such as hepatocellular carcinoma, pancreatic cancer, prostate cancer, osteosarcoma, breast cancer, colorectal cancer, and ovarian cancer, and minimal expression on normal tissues provide a useful therapeutic window.^17^ Enoblituzumab (MGA271) is a humanized, fragment crystallizable (Fc)-optimized anti-B7-H3 mAb, with an Fc region engineered for increased affinity to the activating Fcγ receptor (FcγR)IIIA (CD16A) and decreased affinity for the inhibitory FcγRIIB (CD32B) to potentially enhance Fc-mediated activities including antibody-dependent cell-mediated cytotoxicity (ADCC).^18^ A phase I clinical trial of 179 patients demonstrated that enoblituzumab monotherapy was well tolerated, with no dose-limiting toxicity (DLT) and no maximum tolerated dose (MTD) defined at a dose of up to 15 mg/kg weekly. Disease stabilization (>12 weeks) and tumor shrinkage (2%–69%) were seen across several tumor types, including melanoma, prostate cancer, bladder cancer, breast cancer, clear cell renal carcinoma, NSCLC, and HNSCC.^19^ In addition, a phase II neoadjuvant prostate cancer trial (NCT02923180) demonstrated that CD8 T-cell density in prostatectomy samples was significantly higher in enoblituzumab-treated patients compared with age-matched and stage-matched untreated prostatectomy controls.^20^

We hypothesized that combination therapy with enoblituzumab and pembrolizumab would improve tumor control by targeting two independent checkpoint pathways resulting in coordinated engagement of innate and adaptive arms of the immune system to maximize tumor regression. We conducted a phase I/II study of enoblituzumab in combination with pembrolizumab in patients with advanced solid tumors.

## Methods

### Cell culture

Peripheral blood mononuclear cells (PBMCs) were separated by Ficoll (GE Healthcare) density gradient centrifugation from healthy donor whole blood (StemExpress, Maryland). SAS (human tongue squamous carcinoma cell line) was obtained from Accegen (New Jersey) and maintained in the laboratory. Cells were cultured in RPMI 1640 medium with L-glutamine (Thermo Fisher Scientific) supplemented with 10% fetal bovine serum (Thermo Fisher Scientific), 10 mM HEPES buffer (Sigma-Aldrich), and penicillin-streptomycin (Thermo Fisher Scientific). Recombinant human IL-2 (rhIL-2, Peprotech) was supplemented as indicated.

### Antibody-dependent cellular cytotoxicity

PBMCs (3×10^5^/well) were cocultured with SAS target cells at a 30:1 ratio in the presence of serial titration of enoblituzumab or control mAb in a 96-well plate. Culture supernatant was collected at 24 hours for the measurement of cytotoxicity using CytoTox cytotoxicity assay kit (Promega).

### In vitro PBMC activation

Coculture of PBMCs and SAS target cells (effector:target=20:1) was stimulated with enoblituzumab (0.05, 0.5, 5 µg/mL) alone or in combination with anti-PD-1 (0.2 µg/mL) in culture medium supplemented with 20 µg/mL of interleukin-2 in a 96-well plate. Culture supernatant and cells were collected at different time points for cytokine ELISA and FACS analysis, respectively. At day 6 of culture, cells were restimulated with phorbol myristate acetate/ionomycin using Cell Activation Cocktail (BioLegend) overnight to assess the capacity of immune cells to produce interferon gamma (IFN-γ). Golgistop (BD Biosciences) was added to the culture overnight before the intracellular IFN-γ measurement.

### Flow cytometry

The following antibodies were used: antihuman CD3-FITC or V500, CD4 PerCP-Cy5.5, CD8 FITC or V450, CD56 PE or BV510, IFN-γ APC, PD-L1 APC, and mIgG1 APC isotype control (all from BD Biosciences). Cell surface staining was performed by incubating cells with mAbs for 30 min at 4°C in FACS staining buffer (BD Biosciences) followed by 2 times washing with phosphate-buffered saline. For intracellular staining, surface stained cells were fixed and permeabilized using fixation/permeabilization buffer (Thermo Fisher Scientific) and stained with anti-IFN-γ mAb according to the manufacturer’s instructions. Samples were resuspended in FACS buffer and acquired using a LSRFortessa flow cytometer (BD Biosciences) with FACSDiva software and analyzed using FlowJo software (FlowJo). For FACS data analysis, doublets were excluded using forward scatter height and width properties and dead cells were excluded by FVD780 (Thermo Fisher Scientific) positive staining.

### IFN-γ ELISA

The level of IFN-γ in culture supernatant was measured by ELISA (R&D Systems) following the manufacturer’s instruction.

### Study design and patient population

**Figure 1:**
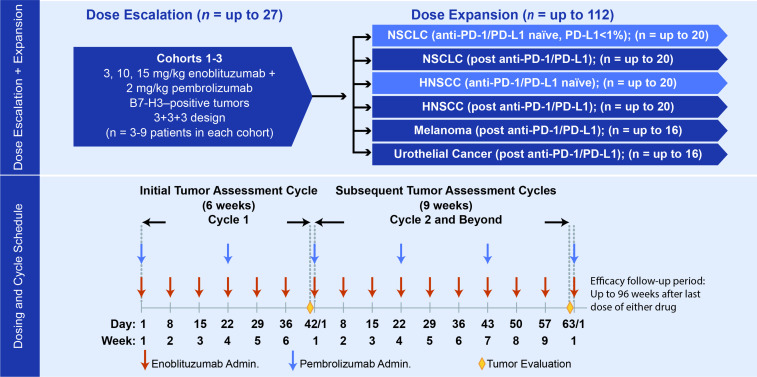
Trial design. HNSCC, head and neck squamous cell carcinoma; NSCLC, non–small cell lung cancer; PD-1, programmed cell death-1; PD-L1, programmed death-ligand 1.

This multicenter, phase I/II, open-label, dose-escalation, and cohort expansion study investigated the safety, pharmacokinetics (PK), pharmacodynamics, immunogenicity, and antitumor activity of enoblituzumab in combination with pembrolizumab in patients with advanced solid tumors. This study enrolled patients with multiple tumor types in a dose-escalation phase to determine the MTD or maximum administered dose (MAD) of the combination, followed by a cohort expansion phase in patients with melanoma, NSCLC, HNSCC, and urothelial cancer (figure 1). The cohorts of patients with HNSCC and NSCLC included patients who had received prior anti-PD-1–containing or anti-PD-L1–containing therapy and patients who had never received prior anti-PD-1–containing or anti-PD-L1–containing therapy. Safety and PK data are summarized for all enrolled patients; preliminary efficacy data focus on patients with HNSCC and NSCLC owing to limited responses in other tumor types.

Patients were required to be ≥18 years of age with histologically proven, locally advanced or metastatic disease that was measurable per Response Evaluation Criteria in Solid Tumors (RECIST) version 1.1. Patients had to have good Eastern Cooperative Oncology Group performance status (0 or 1) and acceptable end-organ function. Disease-specific prior therapy requirements for dose escalation required that patients had disease progression during or following at least one and up to five prior therapeutic regimens. Dose escalation proceeded using a conventional 3+3+3 approach,^21^ and the MTD was defined as the dose level at which <33% of patients experienced a DLT during cycle 1. If none of the first three patients treated at a given dose level experienced a drug-related DLT, three additional patients were to be treated with the higher dose level. If one of the first three patients treated at a given dose level experienced a drug-related DLT, three additional patients were to be treated at the same dose level. If one of these additional three patients (corresponding to two of the six patients enrolled in the cohort) experienced a drug-related DLT, three additional patients were to be treated at the same dose level (for a total of nine patients). If none of these additional three patients (corresponding to one of the six patients enrolled in the cohort) experienced a drug-related DLT, three additional patients were to be treated with the higher dose level. The MTD for MGA271 in combination with pembrolizumab was considered to be exceeded if two or more patients out of the first three patients treated at a given dose level, or at least three of six patients treated at a given dose level, or at least three out of nine patients treated at a given dose level experienced a drug-related DLT, and all subsequent patients were to be treated at the next lower dose level. In the dose-escalation phase, three to nine patients were to be enrolled in each dose cohort. Per protocol, during the dose-expansion phase, up to 16 patients each were to be enrolled into melanoma and urothelial expansion cohorts, respectively, up to 40 patients were to be enrolled into the NSCLC cohorts, and up to 40 patients were to be enrolled into the HNSCC cohorts.

Patients with HNSCC were enrolled in two separate cohorts (up to 20 patients each) based on prior receipt of anti-PD-1/PD-L1 therapy. In each of the two HNSCC cohorts, at least 10 patients were required to be human papilloma virus (HPV)-positive, and patients in both cohorts were required to have experienced disease progression after receiving first-line platinum-based systemic therapy. Patients with NSCLC had to have disease progression after receiving first-line histology-specific platinum doublet therapy. Patients with NSCLC with known activating driver mutations in *EGFR* or *ALK* had to have disease progression after the appropriate targeted therapy. All patients in the HNSCC expansion cohort had PD-L1 tumor expression levels assessed by immunohistochemistry, either on archival or new tissue biopsy samples, but determination of PD-L1 expression was not required prior to enrollment. Patients with NSCLC were enrolled in two separate cohorts (up to 20 patients each) depending on prior receipt of anti-PD-1/PD-L1 therapy. PD-1/PD-L1–naïve patients were required to have a PD-L1 tumor positivity score <1% (Dako 22C3 antibody), assessed prior to enrollment.

**Figure 2:**
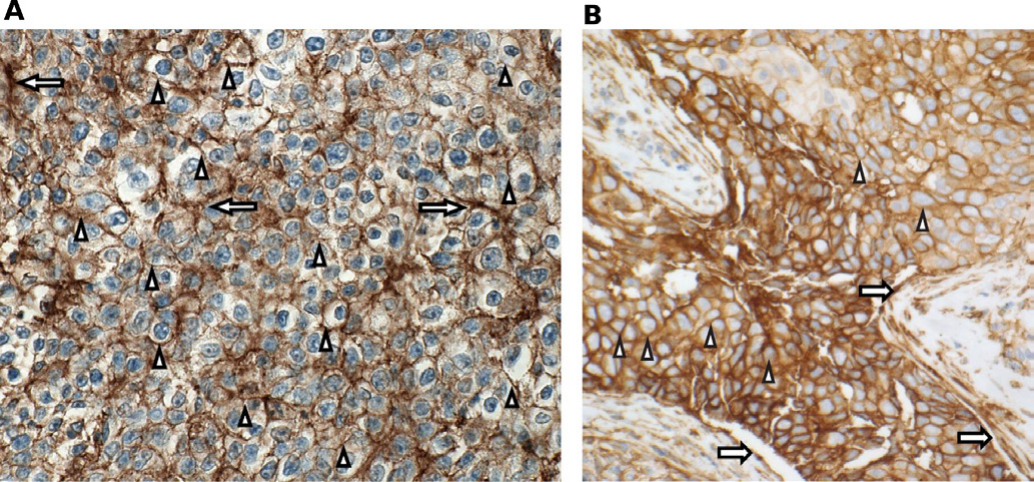
B7-H3 staining. (A) HNSCC PR 2+/3+ membrane/epithelial staining, specifically HNSCC anti-PD-1/PD-L1–naïve patient #17; (B) NSCLC PR 3+ membrane staining, specifically NSCLC anti-PD-1/PD-L1–naïve, PD-L1 TPS <1% patient #12. Arrows indicate endothelial staining; arrowheads indicate membrane staining. B7-H3 positivity was defined as ≥2+ membrane staining (with or without cytoplasmic staining) in ≥10% of cancer cells and/or extensive staining (2+ and/or 3+) in ≥25% of associated tumor vasculature. HNSCC, head and neck squamous cell carcinoma; NSCLC, non–small cell lung cancer; PD-1, programmed cell death-1; PD-L1, programmed death-ligand 1; PR, partial response; TPS, tumor positivity score.

Prospective determination of B7-H3 expression was not required for enrollment in the expansion phase; however, formalin-fixed paraffin-embedded tissue samples or unstained slides were obtained and B7-H3 expression determined by immunohistochemistry staining using a sponsor-developed assay (Covance, Los Angeles, California, USA). B7-H3 positivity was defined as ≥2+ membrane staining (with or without cytoplasmic staining) in ≥10% of cancer cells and/or extensive staining (2+ and/or 3+) in ≥25% of associated tumor vasculature (figure 2).

### Treatment and assessments

Enoblituzumab was administered intravenously over 2 hours once weekly at prospectively planned doses of 3 mg/kg, 10 mg/kg, and 15 mg/kg (MAD in the phase I monotherapy trial) in the dose-escalation phase. During the cohort expansion phase, enoblituzumab was administered at the MTD established from the dose-escalation phase of the study in 6-week cycles during cycle 1, and 9-week cycles thereafter. Pembrolizumab was administered intravenously at a dose of 2 mg/kg over 30 min every 3 weeks, which was the standard dose at the time of study conception. Treatment with the combination could be continued for up to 12 cycles (2 years) or until disease progression, an adverse event (AE) or concurrent illness necessitating discontinuation, withdrawal of consent, protocol non-adherence, or pregnancy.

Safety was monitored from the time of first administration of either study drug through the end-of-treatment visit or 28 days after the last dose of either drug. AEs were graded according to the National Cancer Institute Common Terminology Criteria for Adverse Events version 4.03. irAEs were recorded, and management guidelines were outlined in the protocol, requiring treatment interruption or discontinuation based on grade and duration of the event. Blood samples were collected for determination of immunogenicity (anti-enoblituzumab antibodies) and enoblituzumab PK at specified time points for each cycle.

Concentrations of enoblituzumab in human serum were measured using ELISA with a lower limit of quantification of 62.5 ng/mL. Individual patient PK parameters were derived by non-compartmental analysis using the WinNonlin PK analysis program (Phoenix 64 WinNonlin, version 8.0, Certara, Princeton, New Jersey). PK analyses after the first dose (cycle 1/day 1 [C1/D1]) and after multiple dosing (C2/D1), dose proportionality assessment, and the relationship between total body clearance, volume of distribution at steady state, and serum terminal elimination half-life (*t*_1/2_) and dose were conducted.

Response was assessed by CT or MRI at the end of cycle 1 (week 6) and every 9 weeks thereafter, with a confirmatory scan at a minimum of 4 weeks after the first documentation of a complete response (CR) or partial response (PR). Overall response rate (ORR) and best overall response were determined by conventional RECIST version 1.1 and protocol-defined immune-related response criteria adapted from principles of immune-related response criteria published in 2009^22^; patient management was based on these protocol-defined immune-related response criteria. Duration of response (DOR) was calculated from the time of initial response (CR or PR) documentation (in patients with a subsequent confirmation of objective response) to the time of progressive disease or death, whichever occurred first. Patients were followed for survival up to 96 weeks after the last dose of either drug.

The evaluation of response using immune-related response criteria was based on the following definitions.

#### Immune-related complete response

Disappearance of all target lesions. Any pathological lymph nodes (whether target or non-target) must have reduction in short axis to <10 mm.

#### Immune-related partial response

At least a 30% decrease in the sum of diameters of target lesions, taking as reference the baseline sum diameters.

#### Immune-related progressive disease

At least a 20% increase in the sum of diameters of target lesions, taking as reference the *smallest sum on study* (this includes the baseline sum if that is the smallest on study). In addition to the relative increase of 20%, the sum must also demonstrate an absolute increase of at least 5 mm.

Unlike conventional RECIST criteria, the appearance of new measurable lesions does not automatically denote disease progression under immune-related response criteria. Rather, the dimensions of new measurable lesions are added to the overall sum of tumor diameters for determination of objective response status. Patients will not be considered as having progression unless the new overall sum of diameters has increased by ≥20% from the smallest sum of tumor diameters achieved while on study.

#### Immune-related stable disease

Neither sufficient shrinkage to qualify for partial response nor sufficient increase to qualify for progressive disease, taking as reference the smallest sum of diameters while on study.

### End points

The primary study objective was determination of the MTD or MAD. Efficacy end points included ORR; progression-free survival (PFS; time from the date of first dose until date of documented disease progression or death); overall survival (OS; time from first dose until death); and DOR (time from initial response to progression or death).

### Statistical analyses

Two general populations were used for statistical analysis: the safety population and the response-evaluable population. The safety population comprised all patients who received at least one dose of either enoblituzumab or pembrolizumab. The response-evaluable population comprised all patients who received both enoblituzumab and pembrolizumab and had at least one postinfusion radiographic tumor assessment; only confirmed responses were reported. Patients who received at least one dose of enoblituzumab were included in PK analyses. Two-sided exact CIs were constructed around the ORR. Kaplan-Meier methodology was used to estimate DOR and PFS over time, median DOR and PFS, 3-month and 6-month PFS, and median and 6-month OS. Responders who completed the study without documented disease progression were censored at the date of their last assessment for progression. The method by Brookmeyer and Crowley^23^ was used to construct 95% CIs around PFS estimates of the median and other quartiles for each expansion cohort.

## Results

### Enoblituzumab and anti-PD-1 combinatorial biology

The ability of enoblituzumab to modulate immune responses, including NK cell-mediated ADCC, was assessed in vitro in combination with PD-1 blockade. Consistent with enhanced CD16 engagement, enoblituzumab-mediated ADCC activity (online supplemental figure S1A) was associated with increased interferon (IFN)-γ expression and upregulation of PD-L1 on NK cells (online supplemental figure S1B). Treatment with an anti-PD-1 mAb increased secreted levels of IFN-γ triggered by enoblituzumab (online supplemental figure S1C), enhancing the potential of both NK cells and CD8 T cells to produce IFN-γ following enoblituzumab exposure (online supplemental figure S1C, D). Furthermore, the ADCC potential of effector cells primed by enoblituzumab was enhanced on combination with anti-PD-1 mAb (online supplemental figure S1E). These observations indicate that simultaneous blockade of the PD-1/PD-L1 axis can sustain FcR-mediated immune responses triggered by enoblituzumab, providing mechanistic rationale for the combination approach.

10.1136/jitc-2021-004424.supp1 Supplementary data

### Clinical trial enrollment

Enoblituzumab was administered at doses of 3 mg/kg (n=6), 10 mg/kg (n=3), and 15 mg/kg (n=3) intravenously once weekly in the dose-escalation phase. During the cohort expansion phase, enoblituzumab was administered at 15 mg/kg intravenously once weekly (n=121). Within this report, safety and PK data are provided for all enrolled patients; preliminary efficacy data focus on 37 patients enrolled in the HNSCC cohorts evaluable for response and 35 patients enrolled in the NSCLC cohorts evaluable for response (data cut-off: March 14, 2019). Preliminary response data including the melanoma and urothelial cancer cohorts are also presented along with those of the NSCLC and HNSCC cohorts.

### Baseline patient characteristics

A total of 133 patients were enrolled and included in the safety population. Patients had a median age of 65 years (range, 21–88), with 69.2% male and 88.7% White (table 1).

**Table 1:** Baseline characteristics

Characteristic	Total (N=133)*
Age, years, median (range)	65 (21–88)
Race, n (%)	
White	118 (88.7)
Black	9 (6.8)
Other	4 (3.0)
Asian	1 (0.8)
American Indian/Alaskan native	1 (0.8)
ECOG performance status, n (%)	
0	41 (30.8)
1	91 (68.4)
2	1 (0.8)
No. of prior therapies, median (range)	
All patients (N=133)	2 (0–6)
NSCLC anti-PD-1/PD-L1–naïve† (n=14)	1 (0–5)
NSCLC prior anti-PD-1/PD-L1† (n=21)	2 (0–4)
HNSCC anti-PD-1/PD-L1–naïve† (n=18)	1 (0–3)
HNSCC prior anti-PD-1/PD-L1† (n=19)	3 (0–5)
Urothelial cancer† (n=17)	2 (0–4)
Cutaneous melanoma† (n=13)	3 (0–6)
NSCLC PD-1/PD-L1–naïve cohort, n	14
Histological subtype, n (%)‡	
Squamous	7 (50)
Non-squamous	7 (50)
Prior TKI, n (%)‡	1 (7)

*Of the 133 patients enrolled and treated, 12 were in the escalation phase and 121 in the expansion phase. Overall, there were 16 patients treated in the NSCLC anti-PD-1/PD-L1–naïve cohort, 25 in the NSCLC prior anti-PD-1/PD-L1 cohort, 21 in the HNSCC anti-PD-1/PD-L1–naïve cohort, 24 in the HNSCC prior anti-PD-1/PD-L1 cohort, 21 in the urothelial cancer cohort, 14 in the cutaneous melanoma cohort, and 12 patients with other tumor types.

†Response-evaluable patients. There was a total of 19 patients not evaluable for response, with 2 in the NSCLC anti-PD-1/PD-L1–naïve cohort, 4 in the NSCLC prior anti-PD-1/PD-L1 cohort, 3 in the HNSCC anti-PD-1/PD-L1–naïve cohort, 5 in the HNSCC prior anti-PD-1/PD-L1 cohort, 4 in the urothelial cancer cohort, and 1 in the cutaneous melanoma cohort.

‡Percentages are calculated over 14 total NSCLC PD-1/PD-L1–naïve patients.

ECOG, Eastern Cooperative Oncology Group; HNSCC, head and neck squamous cell carcinoma; NSCLC, non–small cell lung cancer; PD-1, programmed cell death-1; PD-L1, programmed death-ligand 1; TKI, tyrosine kinase inhibitor.

### Pharmacokinetics

**Figure 3:**
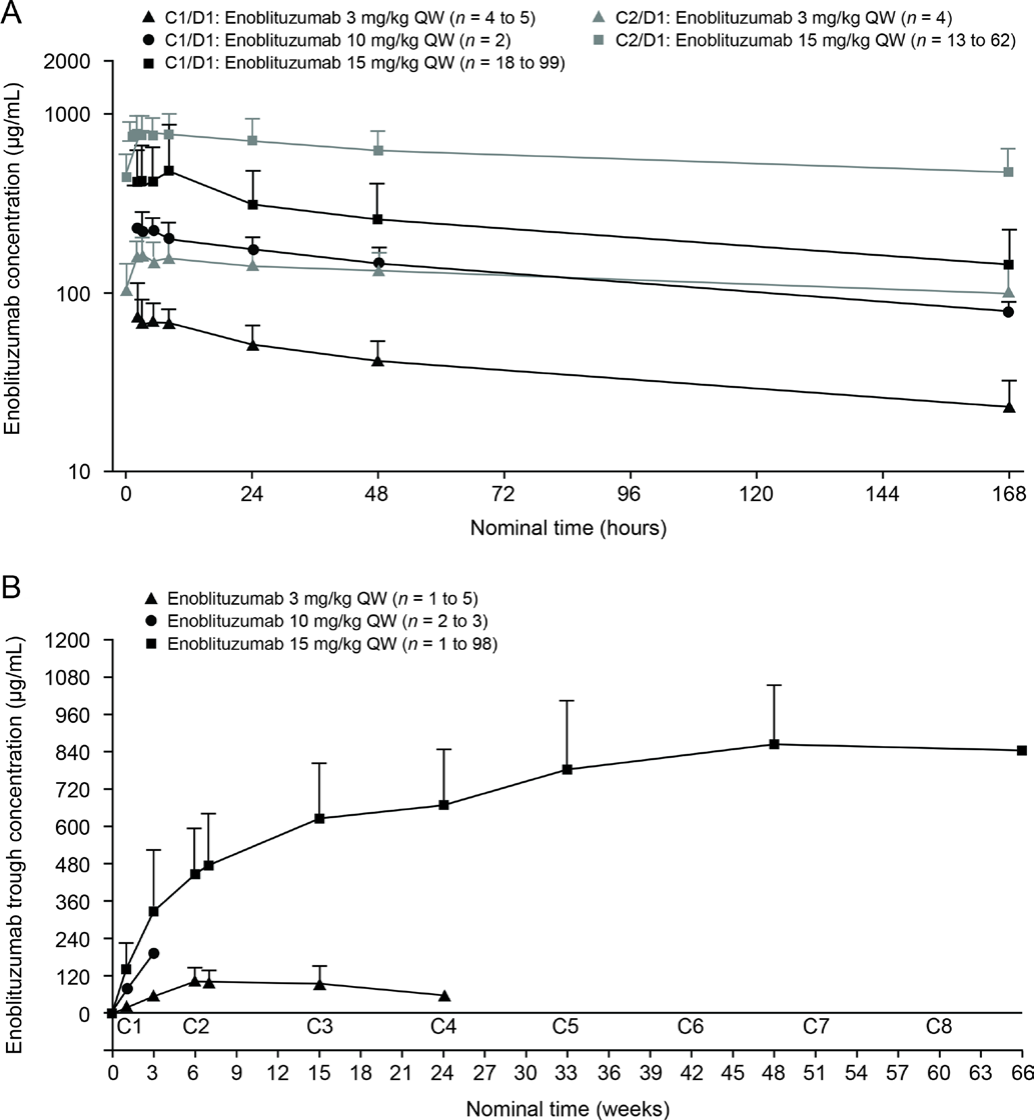
Pharmacokinetics of enoblituzumab after 2-hour intravenous infusions of 3–15 mg/kg QW. (A) Serum concentration–time profiles (semi-log scale). (B) Serum trough concentrations. Symbols and error bars represent arithmetic means and SDs, respectively. The duration of time between C1 and C2 was 6 weeks. C, cycle; D, day; QW, once weekly.

Serum concentration–time data for enoblituzumab were available for 130 patients (n=6, 3, and 121 in the 3, 10, and 15 mg/kg dose groups, respectively), of whom 106 (n=5, 2, and 99, respectively) were evaluable after the first dose (C1/D1) and 66 (n=4, 0, and 62, respectively) were evaluable for PK analysis after multiple dosing (C2/D1). The PK parameters and profiles are presented in table 2 and figure 3, respectively.

**Table 2:** Summary statistics of pharmacokinetic parameters of enoblituzumab after 2-hour intravenous infusion doses of 3–15 mg/kg weekly

Parameter	Unit	Statistic	Weekly enoblituzumab dose		
			3 mg/kg	10 mg/kg	15 mg/kg
First dose, C1/D1					
*C*_max_	µg/mL	GM (%CV) (n)	84 (34) (5)	238 (14) (2)	420 (40) (99)
*t*_max_	hour	Median (min–max) (n)	3 (2–5) (5)	2.5 (2–3) (2)	3 (2–48) (99)
AUC_τ_	µg·hour/mL	GM (%CV) (n)	6254 (35) (4)	21 918 (15) (2)	36 889 (36) (90)
AUC_0–inf_	µg·hour/mL	GM (%CV) (n)	10 321 (46) (4)	36 941 (10) (2)	64 111 (39) (88)
CL	mL/hour/kg	Mean (SD) (n)	0.314 (0.156) (4)	0.271 (0.028) (2)	0.250 (0.087) (88)
*V*_ss_	mL/kg	Mean (SD) (n)	51.9 (14.1) (4)	49.7 (8.8) (2)	46.9 (15.2) (88)
*t*_1/2_	hour	Mean (SD) (n)	128.2 (21.2) (4)	130.7 (9.1) (2)	141.7 (45.6) (88)
Multiple dose, C2/D1					
*C*_max_	µg/mL	GM (%CV) (n)	167 (28) (4)	—	797 (26) (62)
*t*_max_	hour	Median (min–max) (n)	3 (2–8) (4)	—	3 (2–24) (62)
AUC_τ_	µg·hour/mL	GM (%CV) (n)	19 981 (40) (4)	—	93 590 (31) (51)
CL	mL/hour/kg	Mean (SD) (n)	0.160 (0.072) (4)	—	0.168 (0.054) (51)
*V*_ss_	mL/kg	Mean (SD) (n)	59.1 (11.9) (4)	—	62.1 (21.9) (46)
*t*_1/2_	hour	Mean (SD) (n)	283.7 (95.1) (4)	—	275.6 (124.9) (46)
AI AUC_τ_		GM (%CV) (n)	3.20 (9) (4)	—	2.48 (48) (48)

AI, accumulation index; AUC_0–inf_, area under the serum concentration–time curve from time zero extrapolated to infinity; AUC_τ_, area under the concentration–time curve over the dosing interval; C, cycle; CL, clearance; *C*_max_, maximum observed serum concentration; CV, coefficient of variation; D, dose; GM, geometric mean; *t*_1/2_, half-life; *t*_max_, time of maximum concentration; *V*_ss_, volume of distribution at steady state.

Maximum observed concentration of enoblituzumab at C1/D1 (slope, 1.008; 90% CI 0.829 to 1.187) increased in a dose-proportional manner (figure 3A). Systemic exposure in terms of the area under the serum concentration–time curve from time zero extrapolated to infinity (slope, 1.141; 90% CI 0.943 to 1.338) increased in a slightly more than dose-proportional manner over the dose range of 3–15 mg/kg of enoblituzumab. Clearance, volume of distribution at steady state, and *t*_1/2_ were not dose related, suggesting that enoblituzumab exhibits linear PK after the first dose. Following multiple-dose administration, *t*_1/2_ was approximately 12 days, suggesting that enoblituzumab reaches steady state after five half-lives (60 days [approximately 9 weeks]). However, trough serum concentrations over eight cycles for the 15 mg/kg dose continued to increase and approached a plateau by week 33 (figure 3B). This observation implies that the *t*_1/2_ of enoblituzumab would be longer for the 15 mg/kg dose after multiple dosing once every week.

### Safety

TRAEs occurred in 116 of 133 (87.2%) patients, most of which (71.0%) were grade 1 and 2. The most common events were infusion-related reactions (IRRs) (n=72; 54.1%), followed by fatigue (n=37; 27.8%) (table 3). Grade ≥3 TRAEs occurred in 28.6% of patients (n=38), primarily as IRRs (n=9; 6.8%) and increased lipase (n=8; 6.0%). Two patients experienced treatment-related cytokine release syndrome (CRS); one of these patients was hospitalized for a serious grade 3 TRAE of CRS and received vancomycin and tocilizumab to manage CRS, recovered, and ultimately discontinued the study. Overall, TRAEs led to discontinuation in 9.8% of patients (n=13). One treatment-related death due to immunotherapy-induced pneumonitis was reported in a patient with HNSCC. Prior therapy for this male patient included radiation with concurrent cetuximab, methotrexate, paclitaxel in combination with carboplatin, and nivolumab. The patient had three metastatic sites: tonsils, mediastinum, and lung. The patient died 30 days after the last dose of enoblituzumab.

**Table 3:** TRAEs observed in ≥5% of patients

TRAE, n (%)	By dose groups (N=133)			By severity (N=133)		
	3 mg/kg (n=6)	10 mg/kg (n=3)	15 mg/kg (n=124)	All grades	Grade 1/2	Grade ≥3
IRRs	2 (33.3)	1 (33.3)	69 (55.6)	72 (54.1)	63 (47.4)	9 (6.8)
Fatigue	2 (33.3)	1 (33.3)	34 (27.4)	37 (27.8)	34 (25.6)	3 (2.3)
Rash	1 (16.7)	1 (33.3)	13 (10.5)	15 (11.3)	13 (9.8)	2 (1.5)
Nausea	0	0	13 (10.5)	13 (9.8)	13 (9.8)	0
Fever	0	1 (33.3)	11 (8.9)	12 (9.0)	12 (9.0)	0
Lipase increased	0	0	11 (8.9)	11 (8.3)	3 (2.3)	8 (6.0)
Arthralgia	1 (16.7)	0	9 (7.3)	10 (7.5)	10 (7.5)	0
Diarrhea	0	0	9 (7.3)	9 (6.8)	8 (6.0)	1 (0.8)
Decreased appetite	0	0	9 (7.3)	9 (6.8)	7 (5.3)	2 (1.5)
Hypothyroidism	0	0	8 (6.5)	8 (6.0)	8 (6.0)	0
Pneumonitis	1 (16.7)	0	7 (5.6)	8 (6.0)	5 (3.8)	3 (2.3)
Anemia	0	0	7 (5.6)	7 (5.3)	6 (4.5)	1 (0.8)
Pruritus	0	0	7 (5.6)	7 (5.3)	7 (5.3)	0
Lymphocyte count decreased	0	0	7 (5.6)	7 (5.3)	1 (0.8)	6 (4.5)
Chills	1 (16.7)	0	6 (4.8)	7 (5.3)	7 (5.3)	0
**Total patients with ≥1 event**	**5** (**83.3**)	**3** (**100**)	**108** (**87.1**)	**116** (**87.2**)	**78** (**58.6**)	**38** (**28.6**)

IRR, infusion-related reaction; TRAE, treatment-related adverse event.

The most frequently occurring irAE was rash (n=15; 11.3%), followed by thyroid events (eg, hypothyroid, elevated thyroid-stimulating hormone; n=10; 7.5%) and arthralgia (n=9; 6.8%), and most events were grade 1 or 2 (table 4). No MTD was defined; thus, the MAD of 15 mg/kg of enoblituzumab was administered weekly during cohort expansion.

**Table 4:** Immune-related AEs

Immune-related AE, n (%)	All patients (n=133)			PD-1–naïve patients (n=46)		
	All grades	Grade 1/2	Grade ≥3	All grades	Grade 1/2	Grade ≥3
Rash	15 (11.3)	13 (9.8)	2 (1.5)	7 (15.2)	6 (13.0)	1 (2.2)
Thyroid	10 (7.5)	10 (7.5)	0	5 (10.9)	5 (10.9)	0
Arthralgia	9 (6.8)	9 (6.8)	0	3 (6.5)	3 (6.5)	0
Diarrhea	8 (6.0)	7 (5.3)	1 (0.8)	4 (8.7)	4 (8.7)	0
Hepatic	8 (6.0)	8 (6.0)	0	0	0	0
Pneumonitis	8 (6.0)	5 (3.8)	3 (2.3)	3 (6.5)	2 (4.3)	1 (2.2)
Anemia	7 (5.3)	6 (4.5)	1 (0.8)	0	0	0
Lymphopenia	7 (5.3)	1 (0.8)	6 (4.5)	3 (6.5)	1 (2.2)	2 (4.3)
Colitis	3 (2.3)	3 (2.3)	0	1 (2.2)	1 (2.2)	0
Adrenal insufficiency	2 (1.5)	1 (0.8)	1 (0.8)	2 (4.3)	1 (2.2)	1 (2.2)
Myalgia	2 (1.5)	2 (1.5)	0	1 (2.2)	1 (2.2)	0
Myocarditis	2 (1.5)	1 (0.8)	1 (0.8)	0	0	0
Pancreatitis	1 (0.8)	1 (0.8)	0	0	0	0
Thrombocytopenia	1 (0.8)	1 (0.8)	0	0	0	0
**Total**	**83** (**62.4**)	**68** (**51.1**)	**15** (**11.3**)	**29** (**63.0**)	**24** (**52.2**)	**5** (**10.9**)

Immune-related AEs were defined as AEs determined by the investigator to be treatment related and that have been traditionally classified as immune-related AEs in the context of immuno-oncology agents.

No MTD was defined; thus, the MAD of 15 mg/kg of enoblituzumab was administered weekly during cohort expansion.

AE, adverse event; MAD, maximum administered dose; MTD, maximum tolerated dose; PD-1, programmed cell death-1; TRAE, treatment-related adverse event.

### Antitumor activity

#### Head and neck squamous cell carcinoma

**Figure 4:**
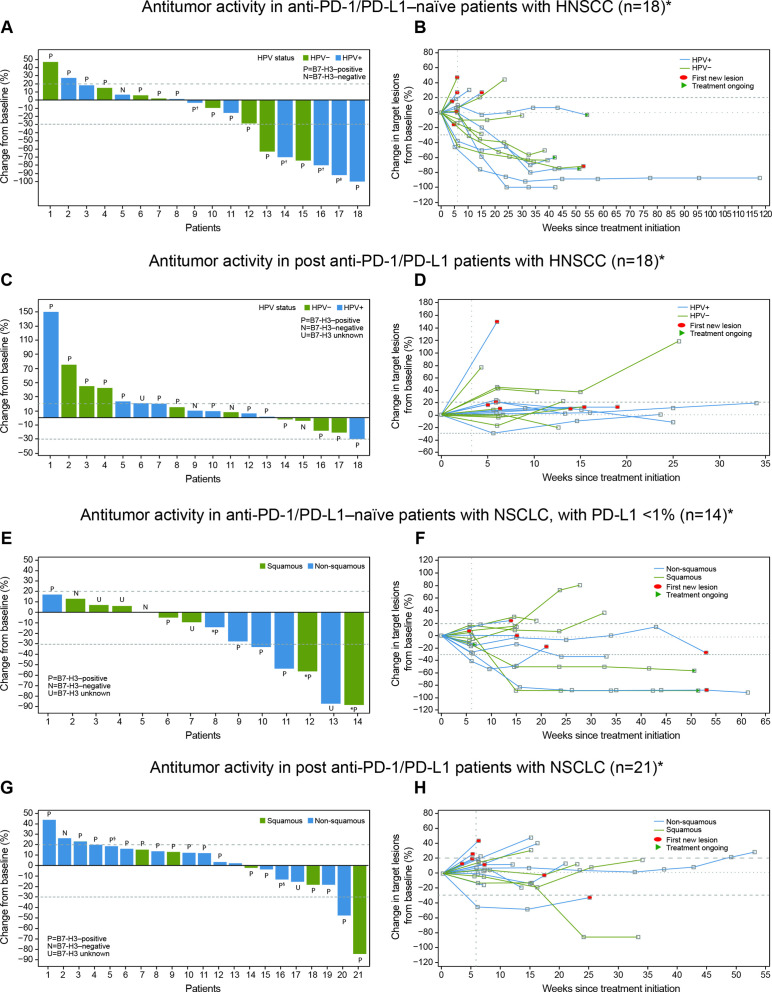
Antitumor activity and DOR in target lesions of response-evaluable patients with HNSCC or NSCLC. The best percent change in target lesion tumor burden from baseline in patients with (A) HNSCC anti-PD-1/PD-L1–naïve, (C) HNSCC post anti-PD-1/PD-L1, (E) NSCLC anti-PD-1/PD-L1–naïve, PD-L1 TPS <1%, and (G) NSCLC post anti-PD-1/PD-L1. The tumor burden (assessed as the longest linear dimension) over time in patients with (B) HNSCC anti-PD-1/PD-L1–naïve, (D) HNSCC post anti-PD-1/PD-L1, (F) NSCLC anti-PD-1/PD-L1–naïve, PD-L1 TPS <1%, and (H) NSCLC post anti-PD-1/PD-L1. B7-H3 expression status at baseline for each patient is indicated (P: B7-H3 positive; N: B7-H3 negative; U: B7-H3 expression status unknown). *Unless noted, patients are in the expansion or dose-escalation cohort at 15 mg/kg enoblituzumab plus 2 mg/kg pembrolizumab. All patients received at least one dose and had at least one postbaseline tumor evaluation. †Treatment ongoing. ‡These patients received 10 mg/kg enoblituzumab+2 mg/kg pembrolizumab. §These patients received 3 mg/kg enoblituzumab plus 2 mg/kg pembrolizumab. HNSCC, head and neck squamous cell carcinoma; HPV, human papilloma virus; NSCLC, non–small cell lung cancer; PD-1, programmed cell death-1; PD-L1, programmed death-ligand 1; TPS, tumor positivity score.

**Figure 5:**
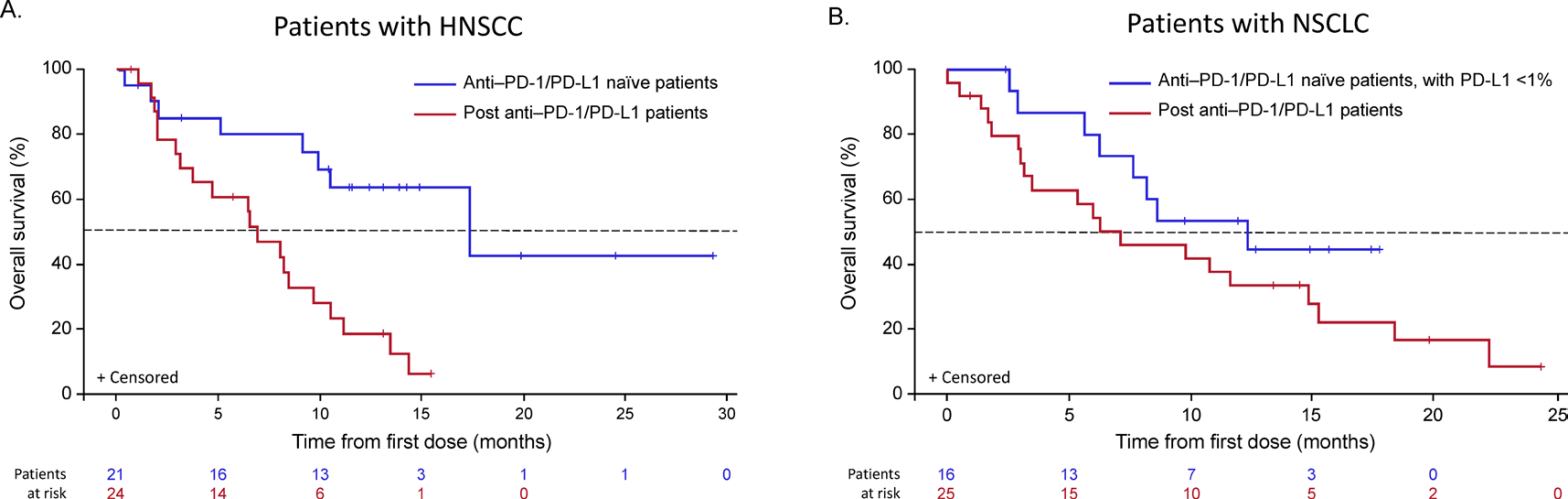
Overall survival Kaplan-Meier curves for patients with HNSCC or NSCLC (safety population). HNSCC, head and neck squamous cell carcinoma; NSCLC, non–small cell lung cancer; PD-1, programmed cell death-1; PD-L1, programmed death-ligand 1.

Of the 21 patients with HNSCC who were anti-PD-1/PD-L1–naïve, 18 were evaluable for response, with an ORR of 33.3% (95% CI 13.3 to 59.0; n=6). Of the 18 response-evaluable patients, 5.6% (n=1) had a CR and 27.8% (n=5) had a PR; stable disease (SD) was observed in an additional 5 patients (27.8%) with a median duration of SD not reached (95% CI 2.1 months to not reached) (table 5). All patients with HNSCC who showed an objective response were B7-H3–positive (figure 2A, —representative image; figure 4). The median DOR was not reached (95% CI 10.7 months to not reached). The 6-month PFS rate was 42.1%, with a median PFS of 3.48 months (range, 0.03–27.1). Ten of 18 patients with HNSCC were HPV-positive, with objective responses in 4 of 10 (40.0%) patients, 3 of whom were PD-L1–positive (PD-L1 status unknown (n=1); online supplemental table S1). Among the HPV-negative patients, the ORR was 25.0% (n=2/8); one responder was PD-L1–positive and one patient’s PD-L1 status was unknown. Of the 24 patients with HNSCC previously treated with anti-PD-1/PD-L1 agents, 19 were evaluable for response and, among these, there were no responses, although 9 (47%) patients experienced disease stabilization, with a median duration of disease control of 3.55 months (range, 2.00–8.05). The range of B7-H3 expression level appeared comparable for both naïve and anti-PD-1/PD-L1 agent experienced across HPV-positive and HPV-negative patients (online supplemental table S1 and figure S2). Antitumor activity in patients with HNSCC is shown in figures 4A–D and 5A.

**Table 5:** Summary of confirmed BOR by RECIST version 1.1

Parameter	HNSCC PD-1/PD-L1–naïve (n=18)	HNSCC prior PD-1/PD-L1 (n=19)	NSCLC PD-1/PD-L1–naïve (n=14)	NSCLC prior PD-1/PD-L1 (n=21)	Urothelial cancer (n=17)	Cutaneous melanoma (n=13)
CR	1 (5.6)	0	0	0	0	0
PR	5 (27.8)	0	5 (35.7)	2 (9.5)	1 (5.9)	1 (7.7)
ORR	6 (33.3)	0	5 (35.7)	2 (9.5)	1 (5.9)	1 (7.7)
SD	5 (27.8)	9 (47.4)	8 (57.1)	11 (52.4)	8 (47.1)	5 (38.5)
Clinical benefit rate*	11 (61.1)	9 (47.4)	13 (92.9)	13 (61.9)	9 (52.9)	6 (46.2)
Progressive disease	7 (38.9)	10 (52.6)	1 (7.1)	8 (38.1)	8 (47.1)	6 (46.2)
NE	0	0	0	0	0	1 (7.7)†
PFS, months						
Median (95% CI)	3.48 (1.35 to NR)	1.45 (1.35 to 3.55)	4.83 (2.60 to 12.22)	3.45 (1.41 to 3.98)	2.18 (1.28 to 5.52)	2.07 (1.31 to 9.82)
Range	0.03–27.1+	0.03–8.05	0.03–12.22	0.03–7.66	0–18.66	0.03–11.96
6-month rate	42.1%	7.5%	43.3%	13.9%	14.0%	25.0%
OS, months						
Median (95% CI)	17.38 (9.17 to NR)	6.93 (3.12 to 9.69)	12.32 (5.65 to NR)	7.13 (3.06 to 14.85)	5.72 (3.09 to 11.1)	14.19 (4.76 to NR)
Range	0.4–29.4+	0.8–15.5	2.4–17.8+	0.03–24.4	0.92–20.4	1.97–18.9+
6-month rate	79.9%	60.9%	80.0%	58.5%	47.6%	78.6%

Data are shown as n (%) unless otherwise noted.

*Clinical benefit rate=CR+PR+SD.

†One patient with cutaneous melanoma has a response as NE.

BOR, best overall response; CR, complete response; HNSCC, head and neck squamous cell carcinoma; NE, not evaluable; NR, not reached; NSCLC, non–small cell lung cancer; ORR, overall response rate; OS, overall survival; PD-1, programmed cell death-1; PD-L1, programmed death-ligand 1; PFS, progression-free survival; PR, partial response; RECIST, Response Evaluation Criteria in Solid Tumors; SD, stable disease.

#### Non–small cell lung cancer

Of the 16 anti-PD-1/PD-L1–naïve patients with NSCLC, 14 were evaluable for response, with an ORR of 35.7% (95% CI 12.8 to 64.9; n=5/14), all of which were PRs; SD was observed in an additional eight patients (57.1%; table 5). The median DOR was 8.3 months (95% CI 2.1 to 8.6), and the 6-month PFS rate was 43.3%, with a median PFS of 4.83 months (range, 2.60–12.22). Of the 25 patients with NSCLC previously treated with anti-PD-1/PD-L1 agents, 21 were evaluable for response and, among these, 2 (9.5%) achieved a PR and 11 (52.4%) had SD, with a median DOR of 3.45 months and a 6-month PFS rate of 13.9%. Among five anti-PD-1/PD-L1–naïve patients, four showed an objective response and B7-H3 positivity and one was non-evaluable, while both patients with prior PD-1 failure who showed objective responses were also B7-H3–positive (figure 2B, —representative image; figure 4). The range of B7-H3 expression was comparable in both PD-1/PD-L1–naïve and prior PD-1 failure patient cohorts (online supplemental table S1 and figure S2). Antitumor activity in patients with NSCLC is shown in figures 4E–H and 5B and prior cancer therapy of the two patients with PR is shown in table 6.

**Table 6:** Prior cancer therapy of the two patients with NSCLC previously treated with anti-PD-1/PD-L1 agents who responded after treatment with enoblituzumab+pembrolizumab

Patient #	Prior regimen	Line of therapy	Duration of treatment (first to last dose)	Number of cycles administered	Best response	Reason for discontinuation
1	**Chemotherapy**					
	Carboplatin/Gemcitabine	1L	120 days (=4 months)	6	Progressive disease	Progression/Recurrence
	Gemcitabine	Maintenance	33 days (=1 month, 2 days)	1	Progressive disease	Progression/Recurrence
	Carboplatin/Pemetrexed	2L	92 days (=3 months, 3 days)	4	Progressive disease	Progression/Recurrence
	**CPI**					
	Nivolumab	3L	570 days (=1 year, 10 months)	40	Stable disease	Progression/Recurrence
2	**Chemotherapy**					
	Carboplatin/Paclitaxel	1L	50 days (=1 month, 22 days)	2	Stable disease	Progression/Recurrence
	**CPI**					
	Atezolizumab	2L	64 days (=2 months, 3 days)	4	Progressive disease	Progression/Recurrence

CPI, checkpoint inhibitor; 1L, first line; 2L, second line; 3L, third line; NSCLC, non–small cell lung cancer; PD-1, programmed cell death-1; PD-L1, programmed death-ligand 1.

#### Urothelial cancer and cutaneous melanoma cohorts

Limited responses were noted in the other cohorts, with one of the 17 patients with urothelial cancer evaluable for response had a PR (ORR of 5.9%; table 5) and one of the 13 patients with cutaneous melanoma evaluable for response had a PR (ORR of 7.7%; table 5).

## Discussion

In this phase I/II, open-label, dose-escalation, and cohort expansion study involving 133 patients, enoblituzumab in combination with pembrolizumab was well tolerated in patients with advanced solid tumors, and the combination was feasible and demonstrated an acceptable safety profile. Most TRAEs were grade 1–2. IRRs were common (54.1%); their mechanism is likely related to the drug molecule itself in that these reactions were usually observed with the first infusion only, quickly resolved, and did not reoccur in subsequent cycles. Grade ≥3 IRRs were seen in nine patients (6.8%), leading to discontinuation in two patients. Thirteen patients (9.8%) discontinued therapy because of TRAEs, including pneumonitis (n=4, including one fatal event), IRRs (n=2), myocarditis (n=2), CRS (n=1), colitis (n=2), adrenal insufficiency (n=1), and pancreatitis (n=1). The most frequently occurring irAE was rash (n=15; 11.3%), most events were grade 1 or 2.

Interestingly, this dual checkpoint-targeted therapy was not associated with an increase in TRAEs or irAEs compared with what is expected for checkpoint-targeted monotherapy, unlike what is observed with combination checkpoint blockade therapies with PD-1 and CTLA-4 inhibition in NSCLC and melanoma.^24^ A pooled incidence of irAEs from combination immune checkpoint therapy^25^ compared with this trial showed an incidence of 32.7% vs 6.8% for all-grade diarrhea, 31.4% vs 5.3% for pruritus, and 27.1% vs 11.3% for rash, respectively. Our preliminary findings suggest that combination therapy with B7-H3 and PD-1 inhibition is feasible with minimal additive toxicity beyond what would be expected with PD-1 monotherapy.

Availability of CPIs has led to a paradigm change in the management of patients with solid tumors. The greatest impact has been observed in patients with HNSCC and NSCLC, where immunotherapy-based regimens are routinely used in the frontline management of metastatic disease. For HNSCC, we now have evidence from the KEYNOTE-048 trial to support the use of pembrolizumab in combination with platinum-based therapy, and pembrolizumab alone for PD-L1 expressing tumors.^6^ While both approaches are preferred over chemotherapy, pembrolizumab plus chemotherapy might be preferred for patients with significant symptom burden and those with a need for objective response. However, this benefit in tumor reduction must be balanced against the incremental toxicities associated with the addition of chemotherapy to pembrolizumab.

In this trial, unlike KEYNOTE-048, the majority of patients with CPI-naïve HNSCC had disease progression after platinum-based chemotherapy. It is therefore noteworthy that combination of enoblituzumab and pembrolizumab resulted in an ORR of 33.3%. This response is numerically higher than previous clinical trial experience with anti-PD-L1 monotherapy regardless of prior platinum exposure for recurrent/metastatic disease.^6 26^ In addition, prolonged SD was seen in most CPI-naïve patients, with an overall clinical benefit rate (CR+PR+SD) of 61.1%.

Similar to HNSCC, there continues to be tremendous interest in improving outcomes in NSCLC and moving beyond PD-1/PD-L1 monotherapy. Combination chemo-immunotherapy is now approved, and routinely used in the first-line setting. There is ongoing interest in developing a ‘chemotherapy-free’ approach that would be equivalent to and perhaps superior to contemporary chemo-immunotherapy regimens. Thus, regimens evaluating combination immunotherapy have been evaluated in clinical trials of NSCLC, and most recently, the US Food and Drug Administration granted approval for use of frontline treatment for nivolumab and ipilimumab in PD-L1–expressing NSCLC.^27^ This combination led to an improvement in OS compared with chemotherapy; it is important to highlight, however, that grade 3–4 TRAEs occurred in 32.8% of patients treated with nivolumab plus ipilimumab and eight treatment-related deaths occurred in the combination immunotherapy arm, reinforcing the need for safer therapies.^27^ In this study, we observed an ORR of 35.7% in patients with CPI-naïve NSCLC with combination enoblituzumab and pembrolizumab.

Although not powered for efficacy, our results demonstrate that dual immunotherapy with the combination of enoblituzumab and pembrolizumab is quite active in patients with CPI-naïve HNSCC and NSCLC. While ORR is not the only indicator of utility of a therapeutic regimen, the findings from this trial provide a provocative signal that needs to be explored further, especially as we evaluate combination immunotherapy regimens to enhance efficacy while mitigating toxicity. A phase II trial of enoblituzumab plus the investigational anti-PD-1 retifanlimab or the investigational anti-PD-1 and anti-lymphocyte-activation gene 3 tebotelimab in HNSCC (NCT04634825) is currently underway.^28^

The observed lack of efficacy in CPI-pretreated patients is not surprising as this subset probably includes patients with more aggressive tumor biology or patients who have developed acquired resistance to PD-1 inhibition. Our study was not designed to understand the biologic basis of immunotherapy resistance but rather to evaluate if there was a signal of efficacy in pretreated disease cohorts. This study does have a number of limitations. This was a small, non-randomized study, that enrolled a relatively small number of patients in disease-specific cohorts. Furthermore, the use of biomarkers for patient selection remains an open question. At this time, it is not clear how to best select patients for enoblituzumab therapy: B7-H3 expression was detected widely across enrolled patients including those demonstrating therapeutic response. In addition, patients on this trial benefited from combination therapy irrespective of PD-L1 expression levels. Moreover, since the study was focused on establishing safety and evidence of therapeutic benefit of enoblituzumab, evaluation of antipembrolizumab antibodies and pembrolizumab PK was not included in the clinical protocol of the study. Further study is required to confirm the preliminary favorable toxicity profile and to eventually compare efficacy and toxicity outcomes to approved immunotherapy combinations in patients with advanced CPI-naïve NSCLC and HNSCC.

## Data Availability

All data relevant to the study are included in the article or uploaded as supplementary information.
