# Supplementary material for: Dual checkpoint targeting of B7-H3 and PD-1 with enoblituzumab and pembrolizumab in advanced solid tumors: interim results from a multicenter phase I/II trial
Source: J Immunother Cancer. 2022 Apr 12;10(4):e004424. doi: 10.1136/jitc-2021-004424 (PMC9006844; doi:10.1136/jitc-2021-004424)
Supplement: Supplementary data [file jitc-2021-004424supp001.pdf]

Journal for ImmunoTherapy of Cancer jitc-2021-002784.R2

SUPPLEMENTAL MATERIAL

Dual checkpoint targeting of B7-H3 and PD-1 with enoblituzumab and pembrolizumab in advanced solid tumors: interim results from a multicenter phase I/II trial

Charu Aggarwal, Amy Prawira, Scott Antonia, Osama Rahma, Anthony Tolcher, Roger B Cohen, Yanyan Lou, Ralph Hauke, Nicholas Vogelzang, Dan P Zandberg, Arash Rezazadeh Kalebasty, Victoria Atkinson, Alex A Adjei, Mahesh Seetharam, Ariel Birnbaum, Andrew Weickhardt, Vinod Ganju, Anthony M. Joshua, Rosetta Cavallo, Linda Peng, Xiaoyu Zhang, Sanjeev Kaul, Jan Baughman, Ezio Bonvini, Paul A Moore, Stacie M Goldberg, Fernanda I Arnaldez, Robert L Ferris, and Nehal J. Lakhani

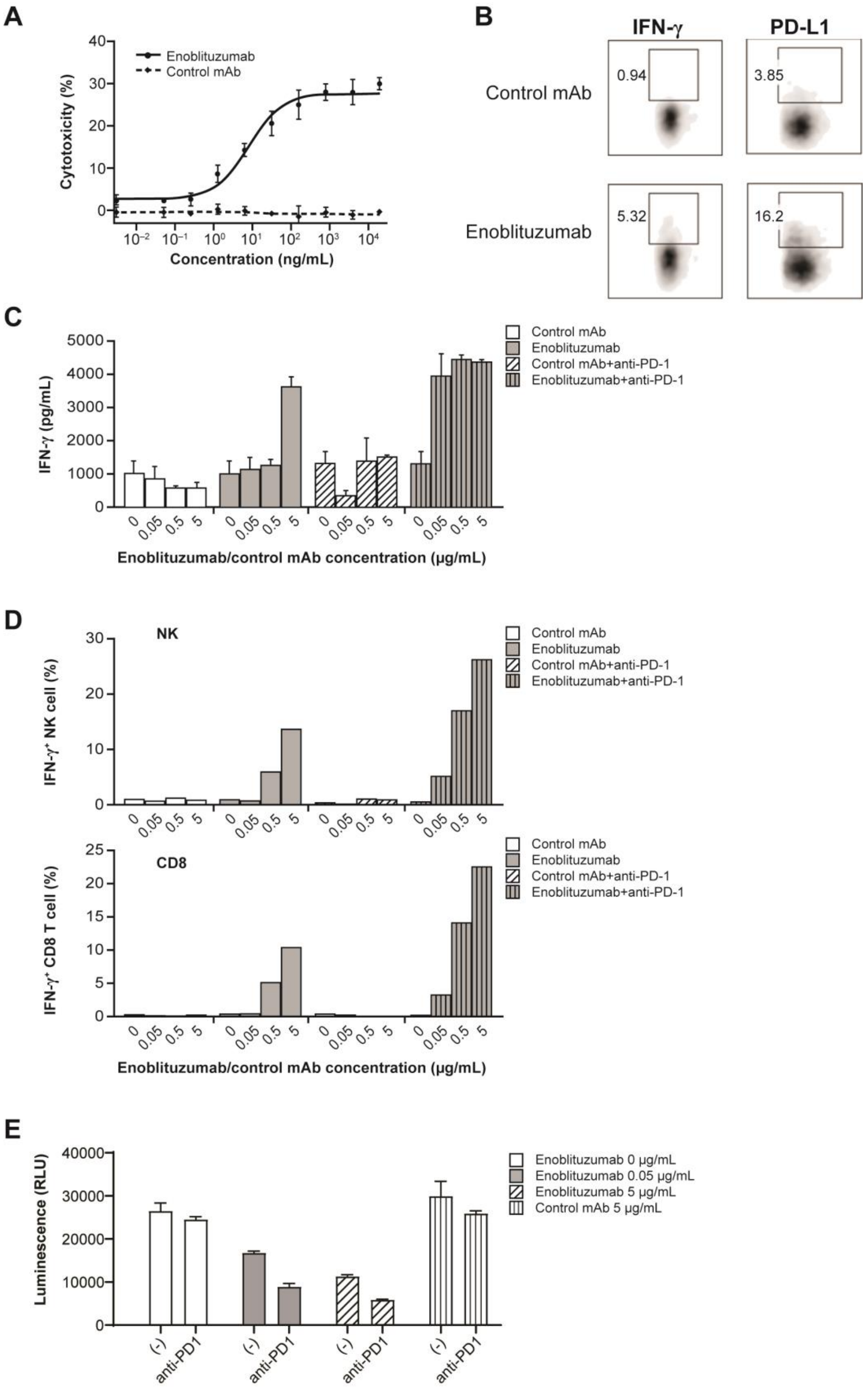
A
Enoblituzumab
Control mAb
Cytotoxicity (%)
Concentration (ng/mL)
B
IFN-γ
PD-L1
Control mAb
Enoblituzumab
0.94
3.85
5.32
16.2
C
IFN-γ (pg/mL)
Control mAb
Enoblituzumab
Control mAb+anti-PD-1
Enoblituzumab+anti-PD-1
Enoblituzumab/control mAb concentration (μg/mL)
D
NK
IFN-γ⁺ NK cell (%)
CD8
IFN-γ⁺ CD8 T cell (%)
Enoblituzumab/control mAb concentration (μg/mL)
E
Luminescence (RLU)
Enoblituzumab 0 μg/mL
Enoblituzumab 0.05 μg/mL
Enoblituzumab 5 μg/mL
Control mAb 5 μg/mL
(-)
anti-PD1

Supplemental figure S1 Anti-PD-1 sustains enoblituzumab-mediated immune responses. (A) Dose-dependent cytotoxicity targeting SAS (B7-H3–positive HNSCC) tumor cells mediated by enoblituzumab. Data shown as mean with SD. (B) Enoblituzumab mediated the upregulation of intracellular IFN-γ (measured at day 1) and surface PD-L1 (measured at day 2) on NK cells from PBMCs cocultured with SAS target cells. The values in FACS plots represent the percent of positive cells within the NK cell gate. (C) The levels of IFN-γ in supernatant from PBMCs cocultured with SAS after stimulation with enoblituzumab alone or in combination with anti-PD-1 mAb for 4 days. Data shown as mean with SD. (D, E) PBMCs were cocultured with SAS in the presence of enoblituzumab alone or in combination with anti-PD-1 mAb for 6 days. (D) Cells were collected and restimulated with PMA/ionomycin in the presence of Golgistop. Levels of NK cell and CD8 T cell specific intracellular IFN-γ was determine by FACS. (E) Alternatively, cells were evaluated for ADCC against the B7-H3 expressing tumor cell line (NCI H1975-luc) at an E:T ratio of 15:1. The loss of luminescence signal was used to measure the target cell lysis. Data shown as mean with SD. Data represent 1 of at least 3 (A, B, C, D) or 2 (E) different experiments using PBMCs from different donors.

Supplemental figure S2 B7-H3 ≥2+ staining levels on tumor cells or vasculature.

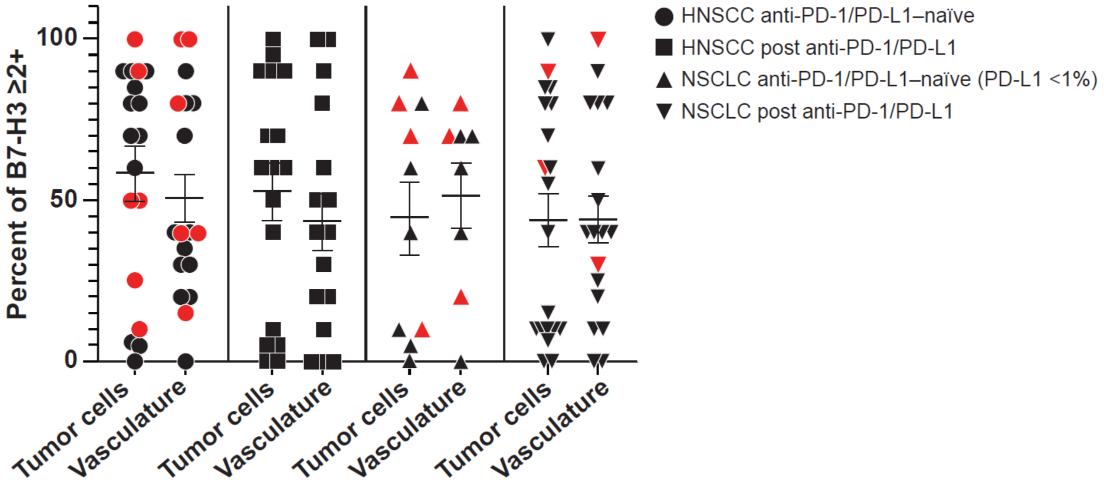

Note: red symbols denote responders (CR and PR). B7-H3, B-cell antigen 7 homolog 3; HNSCC, head and neck squamous cell carcinoma; NSCLC, non–small cell lung cancer; PD-1, programmed cell death-1; PD-L1, programmed death-ligand 1.

BMJ Publishing Group Limited (BMJ) disclaims all liability and responsibility arising from any reliance placed on this supplemental material which has been supplied by the author(s)

Supplemental table S1 Biomarker status of patients with HNSCC or NSCLC

**HNSCC, anti-PD-1/PD-L1–naïve**																		
**Patient number**	**1**	**2**	**3**	**4**	**5**	**6**	**7**	**8**	**9**	**10**	**11**	**12**	**13**	**14**	**15**	**16**	**17**	**18**
**B7-H3**	+	+	+	+	–	+	+	+	+	+	+	+	+	+	+	+	+	+
**B7-H3 (tumor)**	6	5	70	90	0	70	80	85	80	60	90	90	100	90	50	10	50	25
**B7-H3 (vasculature)**	35	80	40	90	0	70	20	30	30	80	40	20	80	100	40	40	100	15
**PD-L1***	–	–	–	+	–	NA	+	–	+	–	+	NA	+	+	–	+	NA	+

**HNSCC, post anti-PD-1/PD-L1**																		
**Patient number**	**1**	**2**	**3**	**4**	**5**	**6**	**7**	**8**	**9**	**10**	**11**	**12**	**13**	**14**	**15**	**16**	**17**	**18**
**B7-H3**	+	+	+	–	+	NA	+	+	–	+	–	+	+	+	NA	+	+	+
**B7-H3 (tumor)**	95	90	100	10	70	NA	60	90	5	40	5	0	60	90	0	70	60	50
**B7-H3 (vasculature)**	100	20	100	20	10	NA	50	0	0	60	0	90	30	40	NA	50	40	80
**PD-L1***	+	+	+	+	+	NA	+	NA	–	NA	+	+	–	+	–	+	–	+

**NSCLC, anti-PD-1/PD-L1 with PD-L1 TPS <1%**														
**Patient number**	**1**	**2**	**3**	**4**	**5**	**6**	**7**	**8**	**9**	**10**	**11**	**12**	**13**	**14**
**B7-H3**	+	–	NA	NA	–	+	NA	+	+	+	+	+	NA	+
**B7-H3 (tumor)**	60	0	NA	NA	5	80	NA	40	10	90	10	80	NA	70
**B7-H3 (vasculature)**	70	0	NA	NA	NA	40	NA	70	60	80	20	70	NA	NA
**PD-L1***	–	–	–	–	–	–	–	–	–	–	–	–	–	–

**NSCLC, post anti-PD-1/PD-L1**																					
**Patient number**	**1**	**2**	**3**	**4**	**5**	**6**	**7**	**8**	**9**	**10**	**11**	**12**	**13**	**14**	**15**	**16**	**17**	**18**	**19**	**20**	**21**

BMJ Publishing Group Limited (BMJ) disclaims all liability and responsibility arising from any reliance placed on this supplemental material which has been supplied by the author(s)

**B7-H3**	+	–	+	+	+	+	+	+	+	+	+	+	+	+	+	+	NA	+	+	+	+
**B7-H3 (tumor)**	10	7	85	80	0	10	70	10	15	10	100	10	40	85	0	80	NA	60	55	90	60
**B7-H3 (vasculature)**	80	10	50	80	25	90	0	10	80	NA	40	20	60	40	40	NA	NA	40	0	100	30
**PD-L1***	–	+	NA	–	NA	+	–	–	–	+	+	–	–	–	+	NA	–	+	–	+	+

*PD-L1 positivity defined as TPS ≥1%. B7-H3, B-cell antigen 7 homolog 3; HNSCC, head and neck squamous cell carcinoma; NA, not available; NSCLC, non–small cell lung cancer; PD-1, programmed cell death-1; PD-L1, programmed death-ligand 1; TPS, tumor positivity score.
